# The Effect of a Taper Angle on Micro-Compression Testing of Mo-B-C Coatings

**DOI:** 10.3390/ma13143054

**Published:** 2020-07-08

**Authors:** Lukáš Zábranský, Katarína Bernátová, Jiří Dluhoš, Rostislav Váňa, Pavel Souček, Petr Vašina, Vilma Buršíková

**Affiliations:** 1 Department of Physical Electronics, Faculty of Science, Masaryk University, Kotlářská 2, CZ–61137 Brno, Czech Republic; kbernatova@mail.muni.cz (K.B.); soucek@mail.muni.cz (P.S.); vasina@physics.muni.cz (P.V.); vilmab@physics.muni.cz (V.B.); 2 Tescan Orsay Holding, a.s., Libušina třída 21, CZ–62300 Brno, Czech Republic; jiri.dluhos@tescan.com (J.D.); rostislav.vana@tescan.com (R.V.)

**Keywords:** micro-compression, micro-pillars, Mo-B-C coatings, stress-strain, taper angle

## Abstract

This research was devoted to studying the influence of the taper angle on the micro-compression of micro-pillars fabricated from near-amorphous and nanocrystalline Mo-B-C coatings. A series of micro-pillars with a taper angle between 4–14° was fabricated by focused ion beam technique. The deformation mechanism was found to be dependent on the taper and, also, on the crystallinity of the coating. In order to obtain correct values of yield strength and Young’s modulus, three empirical models of stress correction were experimentally tested, and the results were compared with nanoindentation measurements. It was shown that the average stress correction model provided comparable results with nanoindentation for the yield strength for taper angles up to ~10°. On the other hand, the average radius or area model gave the most precise results for Young’s modulus if the taper angle was <10°.

## 1. Introduction

The stress–strain behavior is one of the key factors determining the mechanical properties of solids. When the nanoindentation technique is used, it is very complicated to obtain the stress–strain relations due to the constrained flow of material under pressure, leading to a very complex stress field under the indenter depending on its geometry. Micro-pillar compression [1,2,3] has emerged as an alternative technique to analyze the stress–strain dependency of small-scale volumes. In this modification, the sharp Berkovich tip is replaced by a flat-ended tip to probe small cylindrical volumes (so-called micro-pillars) fabricated from the coating to obtain a uniaxial stress–strain behavior, converting the micro-indentation system into a micro-compression system. The micro-pillars are usually fabricated by focused ion beam (FIB) techniques, and their diameters are typically ranging from ~100 nm up to several μm. This technique has already been used to analyze a wide range of materials in either a bulk form or as a thin film [1,2,3,4,5,6,7,8]. However, since this technique is relatively new, there are still no standards for evaluation, and there are several important variables, such as the stiffness and height of the substrate as a part of the micro-pillar, the aspect ratio (height of the micro-pillar divided by its diameter), the taper angle (angle between the pillars wall and central axis), etc., that need to be taken into account. It is very convenient to fix as most of them as possible to avoid any over or under-estimations of calculated mechanical properties. While it is very time-consuming to prepare a large number of micro-pillars to study any possible drawbacks of this technique, the finite element modeling (FEM) [9,10] proves to be very useful for finding general rules how to obtain reliable results from the micro-compression tests. Zhang et al. [9] showed that the aspect ratio in-between 2 and 3 was recommended to eliminate the buckling of the pillar during its compression and that the micro-compression of a pillar with the taper angle of ~3° resulted in an overestimation of calculated elastic modulus due to taper-induced spurious hardening. On the other hand, Fei et al. [10] concluded that the Sneddon’s correction for the compliance [11,12] could be used for obtaining the correct strain for deformation of micro-pillars with high or low aspect ratios and with the taper angle up to 5°, no matter whether the material was plastic or elastic. However, in the stress calculation, it is assumed that the micro-pillar is perfectly uniform throughout its whole height, which is not true if the taper angle is nonzero. Fei et al. [10] proposed several simple stress correction procedures for obtaining the correct stress, although admitting that these corrections were just geometrical rather than having a physical meaning.

This study was devoted to experimentally evaluate the usability of empirical stress corrections theoretically calculated and presented in [10] when applied to micro-compression experiments done to near-amorphous and nanocrystalline Mo-B-C coatings. The Mo-B-C coatings show a very promising combination of high hardness and moderate ductility [13,14,15,16,17,18,19] due to their unique microstructure and chemistry. This unusual combination of high hardness and ductility is very demanding in the field of protective coatings, and a more detailed study of their mechanical properties at micro and nanoscale can give a new insight into how the microstructure affects the properties of coatings for which the micro-compression testing is very suitable. The fabrication of the micro-pillars with the perfect shape is, however, very time-consuming, and each attempt to hasten the process often leads to a higher number of shape imperfections, such as the nonzero taper angle. Finding a proper way how to obtain correct stress during the micro-compression test even for micro-pillars with taper could decrease the experimental time significantly. In this study, the taper angle was varied in the range between 4° and 14° by changing the parameters of the FIB, and from the micro-compression tests, the stress–strain relations were obtained. Afterward, Young’s modulus and yield stress were evaluated and corrected by three empirical models. The values of calculated elastic modulus and estimated hardness were then compared with results obtained by simple quasistatic indentation testing to see which of these three models for stress correction could provide the most precise results.

## 2. Materials and Methods

The Mo-B-C coatings were synthesized by magnetron sputtering of the Mo_2_BC target. The depositions were carried out using a sputtering system with a cylindrical chamber (ø 50 cm × 50 cm). Prior to the deposition process, all cemented tungsten carbide WC-Co substrates were cleaned in an argon plasma (pressure 0.3 Pa) for 20 min. The rotation of the substrate holder was set to 5 rpm during the whole deposition process to ensure good homogeneity of growing films. The deposition pressure was set to 0.1 Pa for all processes.

Two coatings were selected for this study: MoBC_AM_ (Mo/B/C = 40/30/30 at.%) with near-amorphous microstructure and MoBC_CR_ (Mo/B/C = 45/14/41 at.%) with nanocrystalline microstructure. While the MoBC_AM_ sample was prepared by DC (direct current) sputtering (250 W, deposition temperature ~50 °C) [16] without any bias voltage applied to the substrate holder, the sample MoBC_CR_ was synthesized using mid-frequency pulsed DC sputtering (also 250 W and deposition temperature ~50 °C) with a repetition frequency of 350 kHz and off-time of 1 µs and with a bias voltage of –200 V (13.56 MHz). The resulting thickness of the coatings—MoBC_CR_ and MoBC_AM_—was 1.4 μm and 1.8 μm, respectively. Afterward, the as-deposited MoBC_CR_ sample was subjected to thermal annealing under ultra-high vacuum conditions to the final temperature of 1000 °C to promote the crystallinity of the coating. Details of the annealing process are published in [17].

X-ray diffraction was used to determine the microstructure of both selected coatings. The measurements were performed on the Rigaku Smartlab X-ray diffractometer (Rigaku, Tokyo, Japan) with a grazing angle of incidence configuration. The hardness and effective elastic modulus, i.e., Young’s modulus divided by 1 − *ν*^2^, where *ν* is Poisson’s ratio for the tested material, were obtained by depth-sensing nanoindentation performed by Hysitron TI950 Triboindenter (Bruker, Billerica, MA, USA) equipped with a Berkovich tip. The standard procedure proposed by Oliver and Pharr [20] was used for the evaluation of the measured data.

**Figure 1:**
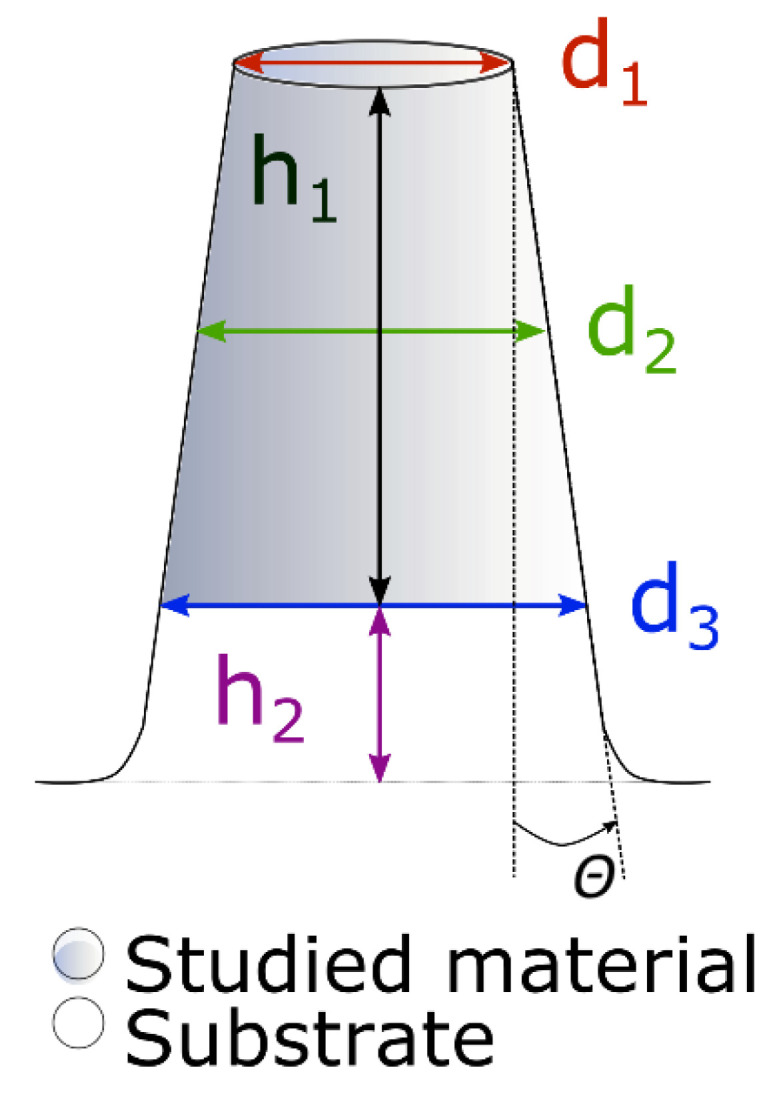
The scheme of a micro-pillar; *d*_1_ corresponds to a diameter of the upper part of the micro-pillar, *d*_2_ is a diameter in the middle of the coating part, *d*_3_ is a diameter of the bottom part of the micro-pillar, *h*_1_ is the height of the coatings part of the micro-pillar, *h*_2_ is the height of the substrate part, and Θ is the taper angle.

The top-down milling method was utilized to produce a set of micro-pillars per sample within a reasonable time (~10 min per pillar). The fabrication of micro-pillars was conducted in Lyra 3 SEM microscope (Tescan, Brno, Czech Republic) with gallium FIB source, and the process consisted of two steps. In the first step, a coarse milling with high ion current (~22 nA, probe aperture 800 μm) was chosen to remove most of the material surrounding the micro-pillar. An annular trench was milled into the sample within multiple passes from outer to inner edge of the trench. The outer diameter was chosen to be 25 μm, so there was enough space for the micro-compression experiment, and the inner diameter was ~5 μm. In the second step, fine milling using a small ion current was utilized in a spiral direction around the pillar. The final diameter of the micro-pillar was reached in only one pass; therefore, dwell time was set to several ms. The exact ion current and the chosen probe aperture during the second step were set according to presets available in the Lyra 3 SEM microscope, and they were varied to produce micro-pillars with different taper angles—pillars with lower taper angle needed to be manufactured with lower ion current and lower aperture. The prepared micro-pillars could be divided into several groups according to their taper angles, and these groups for both samples are summarized in Table 1. The scheme of the final micro-pillar with all useful dimensions can be seen in Figure 1. The aspect ratio (*h*_1_ + *h*_2_)/*d*_2_ (see Figure 1) was fixed to ~2 for all experiments.

The compression of micro-pillars was performed using Hysitron TI950 Triboindenter equipped with a flat punch indenter (20 μm in diameter). The pillars diameter was always lower than the flat punch diameter, so in each measurement, the whole area of the upper part of the pillar was uniformly compressed. The first pillar from each set was tested in a displacement-control regime to find the proper maximum load, causing not only elastic but also plastic deformation. After that, each micro-pillar was tested using quasistatic load function (5 s loading up to this maximum load, 2 s constant load, and 5 s unloading) in a load-controlled regime up to a maximum load of 11 mN. Sneddon’s formula [10,11] for the calculation of compliance associated with the deformation of the elastic half-space was used to obtain the strain of the micro-pillar, assuming that the deformations of the indenter and the substrate were given in terms of their compliance. The system was considered a series of springs consisting of deformations of the indenter Δ*h*_i_, micro-pillar (coating Δ*h*_1_ and substrate Δ*h*_2_ parts), and the substrate Δ*h*_sub_. The total strain Δ*h*_tot_ was then given by:

(1) $$\Delta h_{\mathrm{tot}}=\Delta h_{i}+\Delta h_{1}+\Delta h_{2}+\Delta h_{\mathrm{sub}}$$

The Δ*h*_i_ and Δ*h*_sub_ were given by the Sneddon’s relationship:

(2) $$\Delta h=\frac{\sqrt{\pi }\cdot F\left(1-\nu^{2}\right)}{2\times E\sqrt{A_{p}}}$$

where *F* is the maximum applied force, and *E*, *ν*, and *A*_p_ are respected Young’s modulus, Poisson ratio, and projected area, respectively. The strain of the substrate part of the micro-pillar Δ*h*_2_ could be expressed as:

(3) $$\Delta h_{2}=\frac{Fh_{2}}{\pi E_{s}{}^{2}r_{3}{}^{2}}$$

where *E*_s_ is Young’s modulus of the substrate, and *r*_3_ is the radius of the substrate part of the micro-pillar (see Figure 1). The Young’s modulus of the coating was calculated from the stress/strain ratio from the unloading part of the load-displacement curve; hence, if micro-pillars were with nonzero taper angle, a correction factor for the stress needed to be included. Three empirical corrections based solely on geometry were tested within this study [10]:

(4) $$\sigma_{1}=\frac{F}{\frac{1}{2}\pi \left(r_{1}{}^{2}+r_{3}{}^{2}\right)}$$

(5) $$\sigma_{2}=\frac{F}{\pi {\left(\frac{r_{1}+r_{3}}{2}\right)}^{2}}$$

(6) $$\sigma_{3}=\frac{F}{2\pi }\left(\frac{1}{r_{1}{}^{2}}+\frac{1}{r_{3}{}^{2}}\right)$$

where the *σ*_1_ describes a correction based on averaging the area of the micro-pillar, the *σ*_2_ uses the averaged radius, and the *σ*_3_ stress correction takes the stress averaged from the top and the bottom parts of a micro-pillar.

## 3. Results and Discussion

**Figure 2:**
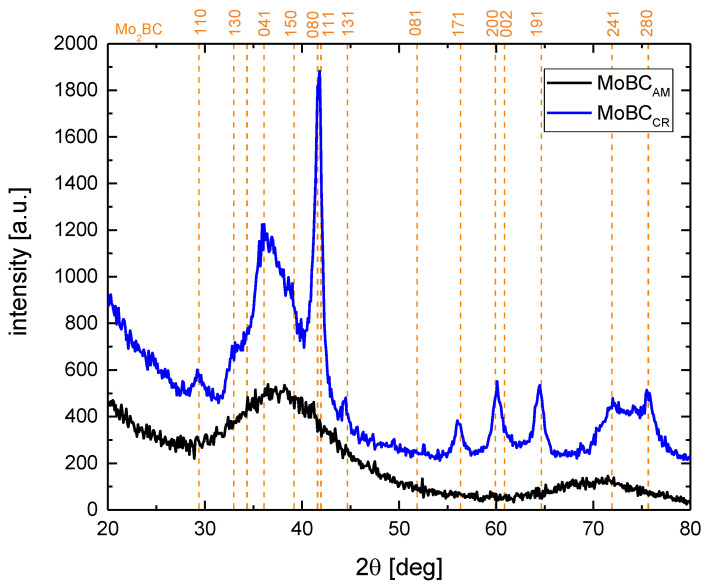
Diffractograms of both studied Mo-B-C coatings with Mo_2_BC reference patterns.

In order to provide a context to the material characterization by compression of micro-pillars, both selected coatings were analyzed by nanoindentation and X-ray diffraction to obtain their hardness and elastic modulus and to evaluate their microstructure. In Figure 2, we could find X-ray diffraction patterns for both Mo-B-C coatings. The diffraction maxima corresponding to selected pronounced Mo_2_BC (COD card no. 5910217) diffractions were plotted with dashed vertical lines for reference. The MoBC_AM_ coatings exhibited a dominant broad peak centered at ~38° and second broad peak with lower relative intensity centered at ~71°. No well-defined sharp peaks implying a crystalline microstructure were observed, and the obtained diffractogram was in accordance with results typical for near-amorphous Mo-B-C coatings [16,17,18]. The diffractogram of the MoBC_CR_ coating showed several sharp peaks with relative intensities and positions corresponding well with the reference Mo_2_BC material. The mean grain size calculated from the observed strongest peak (080), according to the Scherrer formula, was ~12 nm. The hardness and effective elastic modulus were obtained by nanoindentation measurements. The near-amorphous coating showed hardness of ~20 GPa and the effective elastic modulus of ~350 GPa. The crystalline coating showed higher values of both parameters: the hardness of 29 GPa and effective elastic modulus of ~650 GPa, which corresponded to Young’s modulus of 620 GPa, where the Poisson’s ratio was assumed to be 0.26, i.e., the value based on ab-initio calculations [13].

**Figure 3:**
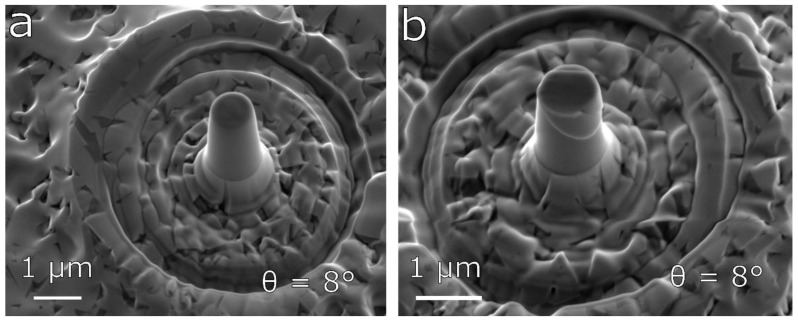
Comparison of fabricated micro-pillars: (**a**) MoBC_AM_ undeformed and (**b**) MoBC_AM_ after compression.

A series of pillars with different taper angles was fabricated from the surface of the MoBC_CR_ and MoBC_AM_ samples. The typical example of undeformed micro-pillar with the taper angle of ~8° for the near-amorphous MoBC_AM_ sample is shown in Figure 3a. Figure 3b shows an image of the corresponding micro-pillar after deformation with the flat punch indenter. We could see that the deformation led to the formation of several shear bands close to the top of the pillar resembling slip traces caused by grain boundary sliding in metals [21,22], albeit, in this case, the coatings posed amorphous microstructure. The sliding mechanism could be explained by the movement of the so-called Somigliana dislocations observed in metallic glasses [23,24]. Under conditions where deformation of amorphous material proceeded through shear localization, an emerging shear band might spread by the propagation of a shear front, which, therefore, represented a kind of dislocation. The stress concentration associated with the shear front could contribute to shear localization. This type of deformation in the case of the MoBC_AM_ sample was observed for both measured taper angles of 4–6° and 7–8°.

**Figure 4:**
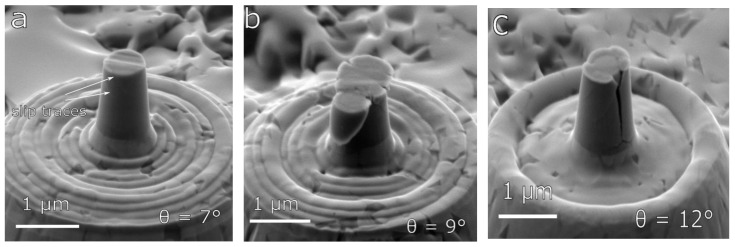
Images of MoBC_CR_ micro-pillars after deformation, showing different deformation mechanisms depending on the taper angle: (**a**) 7° (engineering strain ~0.05), (**b**) 9° (engineering strain ~0.25), and (**c**) 12° (engineering strain ~0.10).

The type of deformation of micro-pillars for the case of the MoBC_CR_ sample depended substantially on the taper angle due to the nanocrystalline microstructure. In Figure 4, we could see images of micro-pillars with different taper angles after compression in a load-controlled regime. The plastic flow in case of micro-pillars with smaller taper angles (≤7°) was localized in the form of small-scale shear bands that were similar to those observed in the sample MoBC_AM_; however, in this case, the coating was composed of nanosized grains, and, thus, the shearing could be explained by grain boundary sliding mechanism that is typical for polycrystalline metallic micro-pillars [21,22]. On the other hand, for higher taper angles in the range between 7° and 12°, the excessive stress concentrations close to the top of micro-pillars resulted in a higher probability of large-scale localized specimen failure. In most cases, the entire top part slipped down, leading to subsequent failure of the whole micro-pillar (see Figure 4b). If the taper angle was higher than 11°, in some cases, the compression was further accompanied by a crack formation, which occurred by through-thickness axial splitting [25,26] in the body of micro-pillars (see Figure 4c). The cracking present for the case of MoBC_CR_ with large taper angles (>11°) was most probably due to the geometry of the micro-pillar and could not be easily claimed as the typical behavior of the material. For taper angles <11°, no brittle failure or presence of crack formation in the body of micro-pillars, i.e., typical behavior for ceramic materials [27], was observed, indicating the expected moderate intrinsic ductility of the Mo-B-C material.

**Figure 5:**
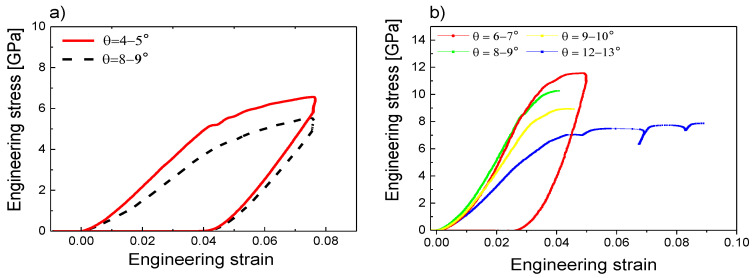
The stress–strain relations depending on the taper angle for (**a**) MoBC_AM_ and (**b**) MoBC_CR_ samples.

The above-mentioned observations for both samples were confirmed by analyzing the stress–strain relations depending on the taper angle, which are presented in Figure 5. Both studied samples first exhibited a linear elastic region for lower strains, and after reaching critical stress, the yielding occurred. The initial part of the elastic region was, however, not precisely linear, which could be caused by the geometry of the top part of prepared micro-pillars. The top edge was not perfectly sharp; it was rather slightly rounded, causing some variance in the pillar’s radius. This nonlinear part of stress–strain relations complicated the later evaluation of Young’s modulus, which had to be extracted from the unloading curves. The actual value of the yield stress depended on the taper angle—for the increasing taper angle, the gradual decrease of the yield stress was observed. For the lowest taper angle, we obtained a compressive yield strength of 10.6 ± 0.7 GPa for the MoBC_CR_ and 5.4 ± 0.5 GPa for the MoBC_AM_ samples. The yield strength for the crystalline sample was comparable with the yield strength of state-of-the-art ceramic-based protective coatings [28,29,30], whereas the resulting yield strength for the amorphous coating reached values typical for diamond-like carbon coatings [29]. While for the amorphous MoBC_AM_ sample, a small-scale shearing was observed, the nanocrystalline microstructure of the MoBC_CR_ sample showed large-scale slipping, which was observed for higher taper angles starting from ~7°.

**Figure 6:**
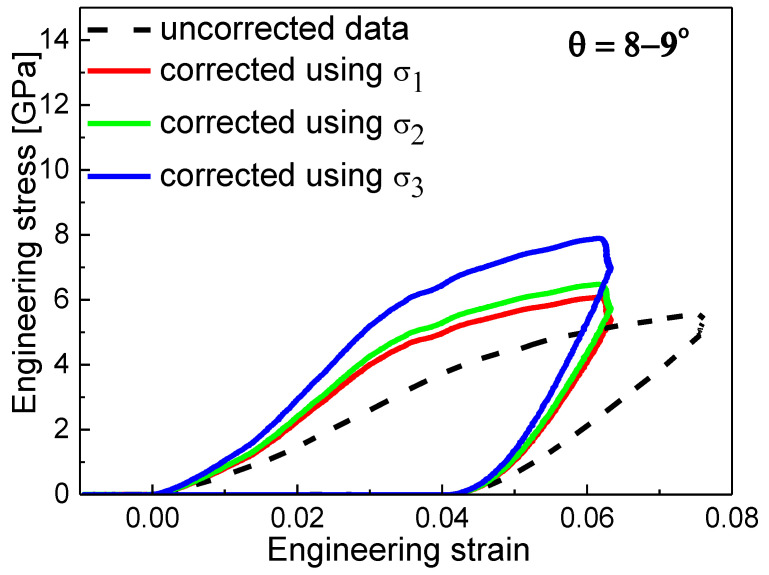
The comparison of uncorrected stress–strain relation and relations corrected by one of the three empirical models for the MoBC_AM_ coating.

The obtained stress–strain relations presented in Figure 5 were without any corrections to the non-zero taper angle. Therefore, the uniform stress was assumed to be present in the whole micro-pillar during the compression test, which was not correct, especially for higher taper, where this simplification could lead to underestimation of the yield strength. The comparison of uncorrected stress–strain relation for the sample MoBC_AM_ (8–9° case) and relations corrected by *σ*_1_, *σ*_2_, and *σ*_3_ models, i.e., averaging the area, radius, or calculated stress at the top and bottom parts of the micro-pillar, is depicted in Figure 6. The uncorrected data line was also without the Sneddon’s corrections for the micro-pillar and the substrate, which was the reason for the shift in strain when compared to corrected data. The corrected data lines for different models differed in stress magnitude significantly, and the different slope of the unloading curves would generate different Young’s moduli.

**Figure 7:**
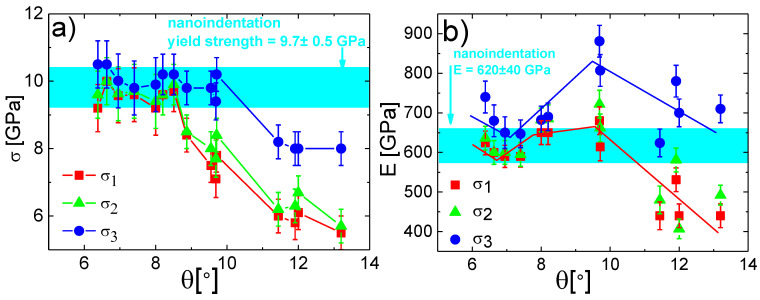
The comparison of (**a**) yield strength and (**b**) Young’s modulus for MoBC_CR_ for different taper angles with the nanoindentation data represented by the cyan region, where its width was given by the experimental error.

**Figure 8:**
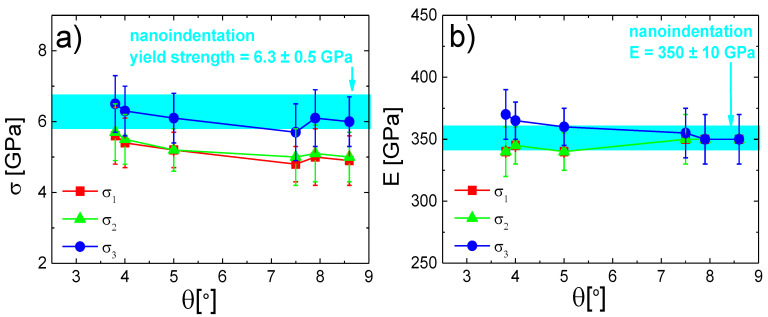
The comparison of (**a**) yield strength and (**b**) Young’s modulus for MoBC_AM_ for different taper angles with the nanoindentation data represented by the cyan region, where its width was given by the experimental error.

To find out which correction model fits best for obtaining correct yield strength and Young’s modulus, the nanoindentation method was used for comparison. The Young’s modulus *E* and hardness *H* were obtained according to the Oliver and Pharr method. The yield strength was approximated by *H*/3 [31]. In Figure 7 and Figure 8, the cyan-colored region represents Young’s modulus and yield strength calculated from the nanoindentation test, where its width was given by the experimental error. The data of yield strength (Figure 7a) and Young’s modulus (Figure 7b) obtained from the micro-pillar compression corrected by one of the three models were plotted as functions of the taper angle for the case of the MoBC_CR_ sample. The corrected yield strength corresponded well with nanoindentation measurements for all three models up to taper angles of 8–9°. For higher taper angles, for the *σ*_1_ and *σ*_2_ corrections, the calculations led to an underestimation of the yield strength, while the *σ*_3_ model resulted in the yield strength that was comparable with nanoindentation results up to ~10°. This conclusion fitted well with theoretical predictions found in [10]. Averaging the stress at the bottom and top parts of a micro-pillar gave the most accurate result of the stress magnitude from all three models. Authors in [10] stated that the accuracy dropped down when the strain was higher than 0.4%, and they modeled micro-pillars with taper angles up to 5°. The results presented in Figure 7a showed that micro-pillars with even higher taper angles could be used for the analysis of yield strength if the averaging of the stress was used for estimation of the stress. From Figure 7b, it was clear that by using the *σ*_3_, i.e., the average stress correction, the obtained Young’s modulus was overestimated even for the smallest taper angles, which also corresponded well with predictions [10]. On the other hand, if the *σ*_1_ and *σ*_2_ corrections were used, the obtained Young’s modulus was within the experimental error of the nanoindentation results for taper angles up to 9–10°. For even higher taper, the values dropped significantly. So, in this case, the averaging of top and bottom radii or area of micro-pillars proved to generate more accurate values of Young’s modulus than averaging of the stress, and the situation was opposite as for the yield strength. This conclusion was not surprising as Young’s modulus in the Sneddon’s equation (see Equation (2)) was dependent on the contact area.

The yield strength and Young’s modulus dependencies for the amorphous MoBC_AM_ sample are plotted in Figure 8a,b, respectively. The evolutions of calculated stresses and moduli using one of the three models were smoother due to the more homogeneous microstructure of the studied material. The corrected yield strength exhibited similar results as in the crystalline case, i.e., the *σ*_3_ average stress correction showed results comparable with the nanoindentation data, whereas the other two models showed underestimation in the calculated yield strength, which increased with the taper angle. On the other hand, perhaps due to a more homogeneous microstructure of the material, Young’s modulus calculated from the compression test resulted in similar results as the modulus obtained by the nanoindentation test for all studied taper angles up to 8–9°. The data corrected by the *σ*_3_ model indicated slightly higher values of *E* than other two models, which was in accordance with findings in the former case; however, they still fitted within the error region, meaning that the results of *E* after correction could be considered as valid up to taper angles of ~8°.

## 4. Conclusions

A series of pillars with different taper angles in the range between 4° and 14° was fabricated from the surface of the near-amorphous and nanocrystalline Mo-B-C coatings, which were afterward subjected to micro-compression testing with the flat punch indenter. In the case of both coatings, the deformation for lower taper angles resulted in the formation of several small-scale shear bands. While this deformation mechanism remained unchanged for higher taper angles up to 9° in case of the near-amorphous Mo-B-C coating, the deformation of the nanocrystalline coating strongly depended on the taper angle. For higher taper angles (7–12°), the micro-compression in case of the nanocrystalline coating led to large-scale failure, where usually the entire top of a micro-pillar slipped down, and for the taper angle >11°, a severe crack formation caused by through-thickness axial splitting occurred in the body of the micro-pillar. The nanoindentation analysis showed the compressive yield strength to be 5.4 GPa and 10.6 GPa for the near-amorphous and nanocrystalline coating, respectively. The yield stress depended on the taper angle—for the increasing taper angle, the gradual decrease of the yield stress was observed.

Afterward, three empirical corrections for stress were applied in order to obtain the correct stress–strain relations for respective micro-pillars with a non-zero taper angle. The calculated yield strength and Young’s modulus were compared with the results obtained by standard nanoindentation measurement. It was experimentally demonstrated that the averaging of the stress at the bottom and top parts of a micro-pillar gave results of yield strength comparable with values obtained by nanoindentation for taper angles up to 10° for the MoBC_CR_ sample and 8° for the MoBC_AM_ sample and, hence, it could be used for correction of the yield strength. On the other hand, the averaging of top and bottom radii or area of micro-pillars showed more accurate values for Young’s modulus up to 9–10° for both studied materials. The main conclusion was that micro-compression testing of pillars with relatively high taper angle produced by fast top-down milling method could still produce reliable results of yield strength and Young’s modulus if the right stress correction was used.

## Figures and Tables

**Table 1:** The dependency of the taper angle on ion current and probe aperture during the second step of the top-down milling.

Sample Name	Ion Current (na)	Probe Aperture (μm)	Taper Angle
MoBC_AM_	0.14	90	4–6°
	2.22	300	8–9°
MoBC_CR_	0.14	90	6–7°
	0.20	90	8–9°
	0.71	200	9–10°
	2.22	300	12–14°
